# pregnancy and weight monitoring: A feasibility study of weight charts and midwife support

**DOI:** 10.1111/mcn.12996

**Published:** 2020-03-28

**Authors:** Julia Sanders, Sue Channon, Rebecca Cannings‐John, Elinor Coulman, Billie Hunter, Shantini Paranjothy, Lucie Warren, Cheney Drew, Bethan Phillips

**Affiliations:** ^1^ School of Healthcare Sciences Cardiff University Cardiff UK; ^2^ Centre for Trials Research Cardiff University Cardiff UK; ^3^ Division of Population Medicine Cardiff University Cardiff UK

**Keywords:** antenatal care, gestational weight gain, motivational interviewing

## Abstract

Around half of pregnant women in the United Kingdom are overweight or obese. The antenatal period provides an opportunity for encouraging women to adopt positive lifestyle changes, and in recent years, this has included development of strategies to support women in avoiding excessive gestational weight gain. The objective of this interventional cohort study was to incorporate individualised gestational weight monitoring charts supported by motivational interviewing (MI)‐based conversations into midwifery‐led antenatal care and assess potential of the intervention for further development and evaluation. The study setting was a community midwifery team within a large maternity unit. The study explored the facilitators and barriers to engagement with the intervention as experienced by women and midwives; 52 women were recruited, of whom 48 were included in the analysis. A single training session was found adequate to prepare midwives to use antenatal weight charts but was insufficient to result in the incorporation of motivational interview techniques into clinical practice. We did not find sufficient evidence to recommend effectiveness testing of this intervention, and there is currently insufficient evidence to support reintroducing regular weighing of pregnant women into UK antenatal care. Given the public health importance of reducing rates of obesity, future interventions aimed at controlling gestational weight gain should continue to be developed but need to include innovative strategies particularly for women who are already obese or gain weight above that recommended.

**Key messages**

- Brief MI training for midwives is insufficient to result in incorporation of discussions of maternal weight into antenatal care.
- Weight charts are acceptable to women but currently lack evidence as an effective method of supporting women achieve a healthy gestational weight gain.
- More complex interventions aimed at supporting pregnant women maintain a healthy weight are required, including interventions appropriate for women who have obesity and pregnant women who gain more weight than recommended.

## 1 INTRODUCTION

Rates of obesity amongst women of child‐bearing age in developed countries have increased steadily since the 1980s, and the public health concern regarding gestational weight gain has shifted from the postwar concern of inadequate nutrition to one of excess weight gain. In the United Kingdom, around half of women commence pregnancy overweight or obese (Euro‐Peristat Project, 2018), with the United Kingdom having the highest rates of maternal obesity in Europe (Devlieger et al., 2016). Women who are obese (with a body mass index [BMI] of 30 or over) are at a greater risk of complications in the antenatal, intrapartum and postnatal periods (Bakun, Karatieieva, Semenenko, Yurkiv, & Berbets, 2018), and excess gestational weight gain is associated with postnatal weight retention (Begum, Colman, McCargar, & al., 2012; Endres et al., 2015) and longer term adverse maternal health effects (Valgeirsdottir et al., 2019).

The development of interventions to help women avoid excessive gestational weight gain is a UK public health priority (National Institute for Health and Clinical Excellence, 2010). Women also expect their weight to be monitored during pregnancy (Daley et al., 2015) and believe it to be beneficial (Allen‐Walker et al., 2017). Global recommended practice on the regularity of weighing pregnant women varies (Scott et al., 2014). Since 1992, US guidance has identified recommended gestational weight gain ranges based on early pregnancy BMI (Institute of Medicine, 1992, 2009) (American College of Obstetricians and Gynecologists, 2013) and encourages regular antenatal weight monitoring. In contrast, in the United Kingdom (National Institute for Health and Clinical Excellence, 2003), regular antenatal weighing is not recommended due to a lack of evidence of effectiveness. Instead, care is focused on providing women with information on the risks of obesity and excess gestational weight gain, together with information on healthy diet and exercise (Denison et al., 2018; National Institute for Health and Clinical Excellence, 2010).

Antenatal interventions targeting diet and exercise in pregnancy have demonstrated modest effectiveness in supporting women to avoid excess gestational weight gain (Muktabhant, Lawrie, Lumbiganon, & Laopaiboon, 2015) and are worthy of further exploration. Weight charts, supported by information on healthy diet and exercise in pregnancy, have been shown to be feasible in the context of US (Aguilera, Sidebottom, & McCool, 2017) and UK (Daley et al., 2015) antenatal care, but a recent high‐quality randomised trial did not demonstrate effectiveness in influencing gestational weight gain (Daley et al., 2019).

A systematic review in a UK service context (Johnson, 2013) highlighted the need for good quality midwifery communication skills in order for a collaborative conversation to take place. Women reported struggling to make sense of the inconsistent and vague information they were given relating to gestational weight gain, and midwives who are themselves overweight may find this as a barrier to them discussing weight with women (Foster & Hirst, 2014).

One approach to enhancing communication is the use of motivational interviewing (MI), a client‐centred approach to communication, which can help engage people with making positive behavioural changes (Miller & Rollnick, 2012). Dealing with ambivalence about behaviour change and building motivation are central to MI, and health care professionals have found it helpful when communicating with obese pregnant women around weight‐related issues (Lindhardt et al., 2015). Brief interventions around weight control have been effective in primary care settings (Aveyard et al., 2016), which have similar time restriction barriers to potential public health interventions as antenatal clinics (Daley et al., 2015).

The existing evidence indicates that further enhancement of interventions is still required to support more women avoid excess gestational weight gain. In this study, we aimed to contribute to this goal by creating an innovative intervention, which combined individualised weight charts with supportive MI conversations with community midwives, focused on monitoring gestational weight in a way that was sensitive to the woman's needs.

### 1.1 Aim

The study's aim was to incorporate individualised gestational weight monitoring charts supported by MI‐based conversations into midwifery‐led antenatal care and assess if the intervention is worthy of further development and evaluation.

### 1.2 Objectives

The study objectives were to

- Develop gestational weight charts informed by UK (Royal College of Obstetricians and Gynaecologists, 2011) and US (American College of Obstetricians and Gynecologists, 2013) guidance,
- Explore the experience of using the charts with women and midwives,
- Assess use of the charts and
- Explore if the intervention warranted further development and evaluation.

## 2 METHODS

### 2.1 Intervention

The intervention combined (a) an individualised weight chart for use by participants at home or in the clinic with (b) support from their community midwife who had received training in an MI approach for engaging in conversations relating to monitoring and managing weight gain during pregnancy. The weight chart intervention was developed in conjunction with a lay advisory group of six recently pregnant women who informed the chart design and content. The study was approved by the National Health Service (NHS) Ethics Committee 16/WA/0221.

Full colour individualised weight charts were produced based on US (American College of Obstetricians and Gynecologists, 2013) recommended gestational weight gain and, for women with an initial BMI of >30, amended to include the contemporary UK (Royal College of Obstetricians and Gynaecologists, 2011) guidance that no gestational weight gain was acceptable. In line with national recommendations, all women at the study site were weighed at their booking appointment (National Institute for Health and Clinical Excellence, 2008) and, in line with local practice, again at 36 weeks gestation.

During work time, participating community midwives attended a group‐based 3‐h face‐to‐face training session from an MI trainer and a study researcher. The session included background to the issue of gestational weight gain, information on the weight charts, the principles of MI, incorporation of MI‐based conversations about maternal weight and gestational weight gain into practice and guidance on plotting maternal weight. Participating community midwives were encouraged to discuss maternal weight at each antenatal appointment, including review of the weight chart, encourage participants to weigh themselves weekly between antenatal appointments and offer to weigh the woman on clinic scales should it be desired by the participant. The MI training of midwives focused on supporting them to engage participants in conversations around weight management in a sensitive and efficient manner. Training on study procedures, but not MI, was provided by the research midwife to hospital‐based antenatal clinic staff who may have weighed study participants during antenatal appointments.

### 2.2 Sample size

An a priori sample size of 50 women was calculated to enable the study to estimate a chart usage rate to 36 weeks gestation of 80% to within 95% confidence interval of ±11%. An estimated attrition rate of 20% to 36 weeks gestation was based on a rate of 6% premature births (Office of National Statistics, 2015) and the remainder being those without a recorded weight at or beyond 36 weeks. A sample of 50 women would allow us to estimate the proportion of women who had gained an appropriate amount of gestational weight at 36 weeks, identified as a primary outcome for a future effectiveness trial.

### 2.3 Recruitment and participation

Women registering their pregnancy with their general practitioner between 27 January and 7 June 2017 and who would receive care from a single community midwifery team received written information about the study when they first attended the surgery.

Women were eligible for inclusion if they were aged over 18 years, had a singleton viable pregnancy of ≤16 weeks gestation confirmed on ultrasound scan, were able to provide informed consent and could communicate clearly in English. Women were ineligible to participate if they were receiving current treatment for a mental health disorder or had an existing medical or obstetric condition that required hospital‐based antenatal care.

Community‐based midwives discussed the study with women at their initial antenatal booking appointment. At around 10–12 weeks gestation, women attended a hospital‐based appointment including an ultrasound scan, baseline weighing and calculation of BMI. Interested women were provided with a further opportunity to ask questions prior to providing written informed consent obtained by a study midwife. An individualised weight chart was printed and incorporated into the woman's hand‐held maternity notes, and women were instructed by a study midwife on its use, including how to record weight on the table and graph.

The weight charts included information on usage. The text suggested participants to record their weight on the chart or table up to once a week. A table was also provided for women to record their weight for later plotting by their midwife. A standard set of bathroom scales was offered to all participants.

### 2.4 Data collection: Quantitative

Once the participant gave birth, the weight chart was copied for analysis, including the number of weight entries and whether the woman, at 36 weeks gestation was within the recommended healthy weight range. The following socio‐economic and obstetric baseline data were extracted from maternity notes of participants following birth: ethnicity, age, gestational age at recruitment, weight, height, BMI, co‐morbidities and parity. Comparative characteristics of all women receiving care at the recruitment site were extracted from the maternity information system.

### 2.5 Data collection: Qualitative

#### 2.5.1 Interviews with women

From 36 weeks gestation, women who had consented to being contacted were invited by phone to participate in an interview to discuss their views on the intervention. Prior to the telephone contact, a midwife researcher checked if the woman had given birth and whether there were any serious pregnancy complications an interviewer should be made aware of. Appointments were made to interview the participant at a convenient time and location. Written consent, including for audio recording and the incorporation of direct quotes in reporting, was obtained prior to interviews. Interviews were conducted either face to face or by telephone by experienced qualitative researchers.

#### 2.5.2 Focus group with midwives

A focus group was conducted with participating community midwives to explore their experiences of the charts and in engaging in MI conversations with women around weight gain in pregnancy, led by an experienced qualitative researcher (SC). Views on the utility, feasibility and acceptability of the weight chart were explored, and any training or preparation needs were identified. Written consent, including for audio recording and the incorporation of direct quotes in reporting, was obtained from midwives before participation.

### 2.6 Data analysis

Quantitative data were analysed using SPSS version 25. Participant characteristics were described using summary statistics: number and proportion and mean alongside standard deviation. Chart usage and the proportion of women reaching 36 weeks gestation within a healthy weight gain was described.

Interviews were transcribed verbatim by independent professional transcribers. The analysis of participant interview data was subjected to thematic analysis and coded, supported by Nvivo software, by an experienced qualitative researcher not involved in data collection (EC). Themes and codes were subsequently collated to form a comprehensive picture of collective experiences and views regarding the intervention. A random selection of transcripts was coded by a second researcher to check for consistency. The analytical process was undertaken in a way that ensured that the integrity of the original transcripts remained intact.

Data from the focus group were subjected to thematic analysis using the same methods and this provided details of midwives' views on the utility, acceptability and impact of the intervention and how the intervention might be adapted for more routine clinical use and any additional training needs required.

### 2.7 Ethical considerations

The PRAM study was approved by the NHS Ethics Committee 16/WA/0221.

## 3 RESULTS

### 3.1 Quantitative results

During the recruitment period, 218 women booked for maternity care with the participating team of community midwives, of whom 52 (24%) were recruited into the study at a mean of 12.3 weeks gestation (range 10–16 weeks, *SD* = 1.2), with a mean BMI of 29.51 (*SD* = 5.13). Table 1 shows the maternal characteristics of the 52 participants at recruitment. One participant was underweight (BMI < 18, 2%); the majority of participants were of healthy weight (BMI ≥ 18 and <25 *n* = 24, 46%) or overweight (BMI ≥ 25 and <30, *n* = 18, 35%); eight participants were obese (BMI ≥ 30 and <40, 15%); and one participant was morbidly obese (BMI > 40, *n* = 1, 2%). Compared with the maternity unit's annual population, the mean age of participants was similar, 31 years (range 18–42), a higher proportion of participants were white and nulliparous, and a lower proportion were obese.

**TABLE 1:** Maternal characteristics of sample

	Study sample *N* = 52	Women booking for maternity care during 2017 at participating hospital *N* = 6,312^a^
Age years, mean (*SD*)	31.2 (5.32)	31.7 (5.76)
Parity (at recruitment) (*n* = 48), *N* (%)		
Nulliparous	26 (54.2)	2,739 (44.1)
Parous	22 (45.8)	3,573 (55.9)
Ethnicity (*n* = 52), *N* (%)		
White	44 (84.6)	4,706 (74.6)
Black/African/Caribbean	0	292 (4.6)
Mixed	2 (3.8)	191 (3.0)
Asian	1 (1.6)	537 (8.5)
Other	5 (9.6)	268 (4.2)
BMI category *N* (%) (*n* = 52)		
Underweight (BMI < 18)	1 (1.9)	75 (1.4)
Healthy (BMI 18 to <25)	24 (46.2)	2,453 (44.4)
Overweight (BMI 25 to <30)	18 (34.6)	1,656 (30.0)
Obese (BMI 30 to <40)	8 (15.4)	1,135 (20.6)
Morbidly obese (BMI 40+)	1 (1.9)	202 (3.7)

Abbreviation: BMI, body mass index.

a Includes some missing data.

All participants received an individualised weight chart and accepted the offer of bathroom scales. The difference between the participants' baseline weight on the clinic scales and on the bathroom scales provided was on average −0.2 kg (*SD* = 0.80, min = −2.5 to max = 1.2 kg).

**FIGURE 1:**
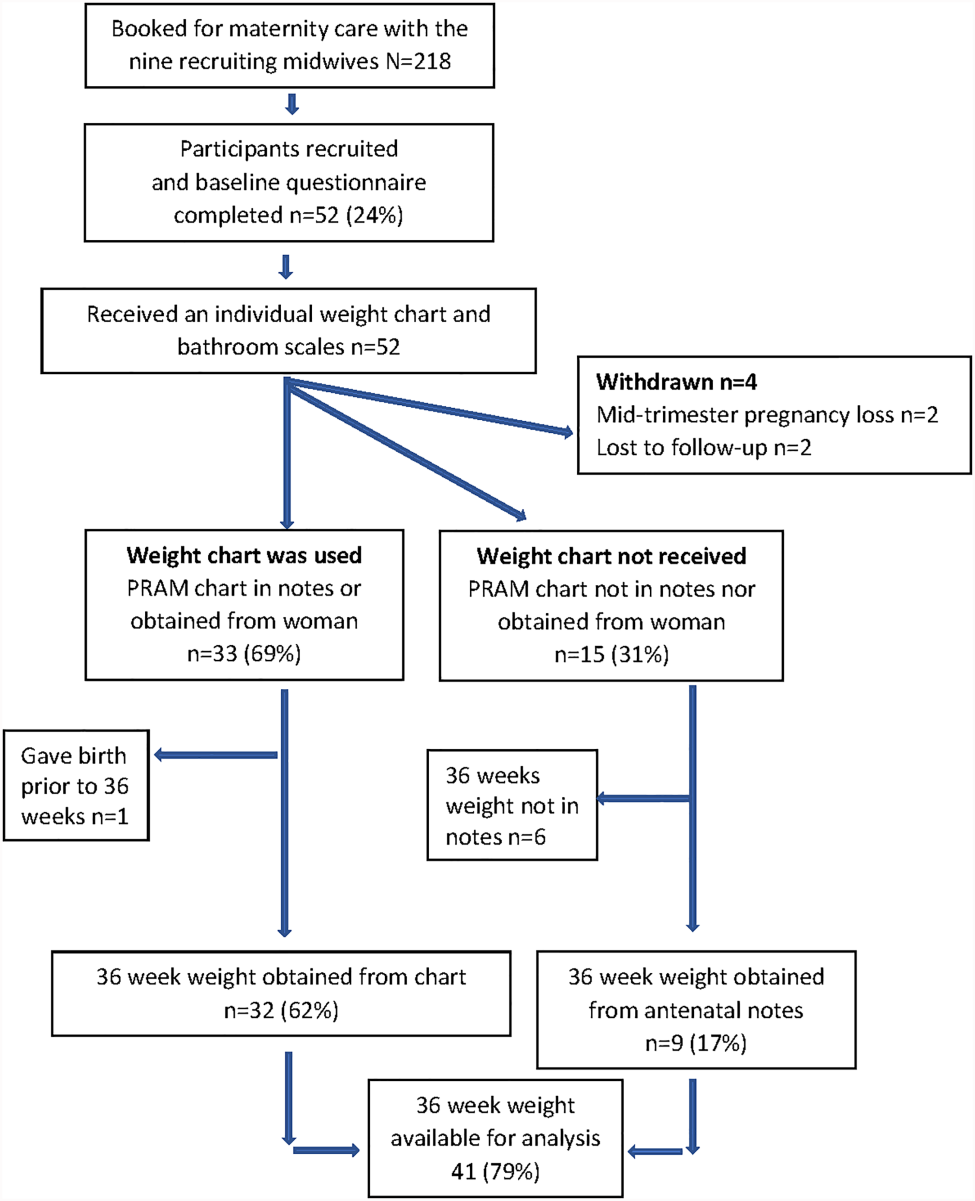
Flow chart of participants

Four women were withdrawn from the study as they moved out of the area (*n* = 2) or due to mid‐trimester pregnancy loss (*n* = 2) (Figure 1). Following study withdrawal, no further data were extracted from the women's maternity notes. Of the 48 participants who were followed up, 33 (69%) participants had the weight chart in their hand‐held maternity notes or it was obtained from the participant following telephone request. The remaining 19 (31%) did not have the study weight chart in their hand‐held maternity notes, nor could it be obtained from the participant herself. Amongst the 33 charts obtained, 31 (94%) had been completed on at least one occasion since booking (28 of the charts [85%] had 10 or more weights plotted) and all had used the table at least once. Two participants used the table but not the chart.

#### 3.1.1 Gestational weight gain

A 36‐week weight was available for 41 women (87%). Amongst the 41 women for whom a late pregnancy weight was available, 17 (42%) were within the recommended health weight range, 20 (49%) had a weight above the healthy range and 4 (11%) had a weight below the healthy range (only applicable for women with a BMI under 30). A higher proportion of women who were obese or overweight in the first trimester had a gestational weight gain above that recommended compared with women commencing pregnancy with a healthy weight, 50.0%, 62.5% and 41%, respectively (Table 2).

**TABLE 2:** Characteristics of women by gestational weight gain at 36 weeks

	Below recommended parameters, *n* = 4/35^a^ (11.4%)	Within range, *n* = 17 (41.5%)	Above recommended parameters, *n* = 20 (48.8%)
Age years, mean (*SD*)	31.08 (2.88)	29.77 (5.75)	32.16 (5.15)
Ethnicity, *N* (%)			
White British	4 (100.0)	13 (76.5)	17 (85.0)
Other	0 (0.0)	4 (23.5)	3 (15.0)
Gestation at recruitment weeks mean (*SD*)	12.33 (1.53)	12.22 (1.09)	12.40 (1.17)
BMI at recruitment on antenatal clinic scales (kg/m^2^)			
Underweight (BMI < 18)	1 (50.0)	1 (50.0)	0 (0.0)
Healthy (BMI 18 to <25)	3 (18.0)	7 (41.0)	7 (41.0)
Overweight (BMI 25 to <30)	N/A	6 (37.5)	10 (62.5)
Obese (BMI 30+)	N/A	3 (50.0)	3 (50.0)
Outcomes			
Birthweight (g), mean (*SD*)	3,303.5 (447.4)	3,544.4 (472.6)	3,725.0 (487.3)
Gestation at delivery (weeks), mean (*SD*)	39.5 (1.29)	40.12 (1.22)	40.00 (1.08)

*Note.* All values are *N* (%) unless otherwise stated.

Abbreviation: BMI, body mass index.

a Only applicable for women with a booking BMI < 30.

### 3.2 Qualitative findings

Individual interviews were conducted with 15 participants, at which time no new issues were being raised and no further participants were invited to interview. Participants were satisfied with study information and appreciated the relaxed approach taken by recruiting midwives. Women cited several reasons for taking part: self‐interest in their gestation weight gain, altruistic reasons, the lack of burden involved and the incentive of weighing scales.

### 3.3 Weight management

Women were asked about gestational weight gain including motivators and the barriers that restricted healthy eating and exercise. Some participants chose a healthy diet during pregnancy to provide the baby with the ‘best start in life’. Other participants were motivated to limit risk factors for pregnancy‐related conditions such as gestational diabetes and to be within eligibility criteria for a midwife‐led birth centre. Others expressed a fear of gaining excessive gestational weight gain due to a history of struggles with personal weight.

Yeah I was more concerned, because I just wanted to be healthy for him. (20)

I was worried about getting gestational diabetes, because my father's got diabetes and because of my BMI I was worried that I might get that… (25)

Some of my friends they put on like three, four stone when they were pregnant and I just kept thinking, I'm at my heaviest now, I can't be that big, because then it wouldn't be healthy for me or the baby. (26)

I found I was putting on more weight towards the end. (25)

But were you able to maintain it to the level that you could still go under midwifery led care? (Interviewer)

Yeah, because I wanted to stay with midwifery and I knew that was a focus for me. (25)

Some women expressed shock that the recommended weight gain in pregnancy was lower than they had expected.

It (recommended weight gain) is, much less than you imagine, even though I'm aware that you don't eat for two. (18)

Factors limiting healthy eating and exercise during pregnancy included nausea or other symptoms and social events.

… there have been times in my pregnancy where I felt so awful and tired and sick and just nauseous all the time … ..I just ate anything I could to make myself feel better and I really didn't care what the weight did. (18)

I went on holiday at week 19, we went all‐inclusive so I made the most of that. (14)

I've got arthritis, and I've had a hip replacement, so I struggle with exercise anyway, so I don't do an awful lot of exercise, because then I'd be crippled the next day. (26)

#### 3.3.1 Practicalities of using the chart

Most women valued the *visual* aspect of the chart, but some experienced difficulties in accurately marking their weight and others would have preferred the charts to have used imperial rather than metric units.

The table was good to plot it, but I found the chart really helpful to be able to see like the increases or the decreases like how steep it had gone up, or how like … how it hadn't maybe gone up and plateaued a bit. (23)

Sometimes I used to plot it in the wrong place, because it's quite small the lines, I'd have to like get another pen to do it. (26)

I am used to stones I would have preferred it in stones. (51)

#### 3.3.2 Chart as a motivator

The extent to which the weight chart acted as a motivator or reassurance varied between women, some stating that study participation impacted their health behaviours and others using the information to inform family members.

I was more careful in what I was eating, because I didn't want to put on a lot in a week and I didn't want to see myself going higher and higher and higher, so yeah definitely, I was definitely more mindful about what I was eating. (93)

And when I've got my mum nagging me, telling me, “Oh, you shouldn't eat that, you're going to put on weight,” I can say, “Well actually, I don't think I'm putting on an unreasonable amount of weight.” (49)

Women suggested that in addition to monitoring weight gain on the chart, incorporating it into an app or providing additional support, such as a physical activity intervention, would be beneficial.

I guess I would have been interested if there had been some classes or something I probably would have come along for a bit more physical activity. (18)

I think when it comes to mapping it on the chart, if it was done in an app or online, that would be quite nice. Because it would be that bit easier than having to kind of map it out. (08)

#### 3.3.3 Weight management as a component of antenatal care

Although some midwives monitored whether participants were weighing themselves and recording their weights on the study charts, discussions regarding gestational weight gain in pregnancy appeared to be initiated by the participants or prompted when women were weighed as part of routine antenatal care at 36 weeks gestation. According to the women, most midwives appeared not to assume their study role in initiating discussions regarding weight gain. Of the discussions that women reported, which followed excess gestational weight, midwives appeared to take on a reassuring rather than advisory role.

How's it going with the PRAM study? Are you still weighing yourself? …So she's asked me those questions but she hasn't really discussed weight with me. (08)

I asked her like what is the, normal weight gain that they expect, [okay]. And she said ten kilos. And I said how that's tiny, [yes] and I said so I've put on almost double that. And she said, yes but it's fine you're healthy and everything is alright, [yes] and she reassured me a lot, [okay] about that weight gain, [right] but that was the first time that it had ever really been mentioned. (18)

### 3.4 Midwives' views

To explore midwives' experiences of the intervention, a focus group was conducted with six of the nine of the midwives, who were able to attend at the prearranged time. An additional midwife, who was unable to attend the focus group, was interviewed separately.

Midwives found the study procedures generally straightforward. Several explanations were given for women not wishing to participate including potential anxiety relating to regular weighing, previous or existing obesity and time constraints.

I think because it was so simple that's why it worked so well. (M1)

I found, you know, larger ladies, I personally didn't recruit anybody. It's like they just didn't, they didn't want to acknowledge that, that they might have a higher BMI to start with. (M4)

The two ladies that I can remember saying no, … I've got 5 kids. (M1)

The study training involved an MI session. Feedback on the session was varied including a view that the session was too short and did not take account of the experience and skill set that midwives will already have as part of their role.

I felt that it was good, because I think it gave you those sort of how to answer and ask those awkward questions, … I found it quite helpful. (M4)

I don't think it was very useful to be honest … It's part of our job anyway isn't it? (M3)

#### 3.4.1 Weight management as a component of midwifery care

Midwives expressed that, prior to the study, they did not have gestational weight gain recommendations and discussed weight only if raised by the woman.

We didn't really have any proper guidelines to how much weight they should put on, or what was acceptable did we. … unless somebody specifically said I think I've put on a lot of weight, I didn't really bring it up again. (M2)

Yeah, so you talk to them about weight and exercise and things like that, but we never really used to say you should only put on so much weight, or you shouldn't put on any more. (M4)

Several midwives expressed that when weight gain differed from the recommended target, anxiety and worry was created for some participants.

… she was like weighing herself all day, like a few times. (M3)

I can't remember what the difference was but here was over the top line and she was basically anxious about that. (M2)

Midwives expressed how when a woman was oedematous they attributed weight gain to fluid retention.

Sometimes it just creeps up a little bit and every time they come in they say oh my ankles are a little bit swollen but everything else is fine, but all the evidence says you can go up a shoe size during pregnancy, so things increase but it's not detrimental to the pregnancy. if you've got somebody who's got gross oedema, their weight gain could be increased, rather than just weight, it's fluid retention. (M1)

## 4 DISCUSSION

This study explored the incorporation of brief but focused MI‐based communication and individual weight charts into midwifery‐led antenatal care. Most women engaged well with self‐weighing, suggesting that this could be incorporated into antenatal care for interested women. Study participation was lower than in a previous similar study (Daley et al., 2015) possibly due to the inclusion of obese women who midwives reported were less willing to enrol. As women who commence pregnancy obese are at greater risk of excessive gestational weight gain and postnatal weight retention compared with women with a healthy BMI (Begum, Colman, McCargar, & al., 2012), interventions that are particularly acceptable to this group need to be developed.

The intention of incorporating the weight chart into each woman's antenatal notes and providing midwives with MI training was to facilitate dialogue about weight gain between the woman and midwife during antenatal check‐ups. Although midwives expressed confidence in discussing weight gain with women, the women's interviews suggested that it rarely happened and there was little evidence that the limited MI training influenced the practice of midwives. A previous trial of antenatal weight charts (Daley et al., 2019) instructed midwives to reset weight gain limits if a woman gained more than recommended, and although this instruction may provide a basis for structured dialogue, there was no evidence that it reduced the risk of women gaining excess weight. In our study, women reported that discussions around maternal weight were usually restricted to information about the need for additional antenatal care or occasions when weight was raised as an issue by the woman or would impact on planned place of birth. When excess weight gain was evident, midwives were found to provide reassurance to women, or attribute this to developing oedema, rather than opening a discussion on weight management.

Similar to other studies (Begum et al., 2012; Daley et al., 2015), we found that nearly half of women gained weight in excess of that recommended. Higher maternal gestational weight gain has received attention as a potentially modifiable factor that increases pregnancy complications including pre‐eclampsia and caesarean section (Flick et al., 2010). As oedema‐related weight gain may occur prior to other signs of pre‐eclampsia (Hillesund et al., 2018) and effective options for weight control in pregnancy remain limited, it was understandable that midwives were inclined to attribute excess weight gain to water retention rather than address a woman's diet and exercise. To date, there have been four trials that have incorporated regular antenatal weighing with components of health provider‐delivered behavioural interventions, which have failed to demonstrate effectiveness (Brownfoot, Davey, & Kornman, 2016; Fealy et al., 2017; Jeffries, Shub, Walker, Hiscock, & Permezel, 2009; McCarthy et al., 2016). Future randomised trials need to incorporate other potential ways of making midwifery‐led discussion around antenatal weight more effective. Also, to increase confidence to start weight‐related discussions, midwives need ways to identify excess fat gain separate from oedema (Widen & Gallagher, 2014) and access to evidence‐based interventions to recommend to women gaining above that recommended.

Weight management, both within and outside of pregnancy, is multifactorial (National Institute for Health and Clinical Excellence, 2010), and it is likely that having a range of options available to woman to provide support prior to, during or following birth may have the greatest overall effect in reducing pregnancy‐related excess weight gain on a population level.

This study had important limitations: the 3‐h MI training session was designed to be deliverable within an NHS service, represented minimal training on the subject and may have been ineffective for this reason. Although the weight chart was incorporated into the notes of participants at recruitment, some participants opted to remove the chart for home use, reducing the opportunity for review and discussion of weight gain by midwives during antenatal check‐ups.

## 5 CONCLUSION

We found antenatal weight charts to be acceptable to women, but a single MI training session was insufficient to result in the incorporation of motivational interview techniques into antenatal care. Previous trials with differing approaches to the use of gestational weight charts have not demonstrated effectiveness (Brownfoot et al., 2016; Fealy et al., 2017; Jeffries et al., 2009; McCarthy et al., 2016), and results of this study did not suggest that the brief MI training provided to midwives, when added to the intervention of gestational weight charts, would be sufficient to yield more positive results. Although there is currently insufficient evidence to support reintroducing regular weighing of pregnant women into UK antenatal care, given the public health importance of reducing rates of obesity, future interventions aimed at controlling gestational weight gain should continue to be developed. Future interventions should include innovative strategies for women who commence pregnancy obese or gain weight above that recommended.

## CONFLICTS OF INTEREST

The authors declare that they have no conflicts of interest.

## CONTRIBUTIONS

JS, SC, RC‐J, BH, SP and LW were involved in the conception and design of the study JS, SC, EC and CD acquired the data. JS, SC, BH, SP, LW, EC and BP analysed and interpreted the data. JS, SC, EC and RC‐J drafted the article. JS, SC, EC, RC‐J, BH, SP and LW revised the article critically for important critical content. JS, SC, RC‐J, BH, SP, LW, EC and CD agreed to be accountable for all aspects of the work. BP provided lay input and advice throughout the study.
